# HPV Vaccine Attitudes and Practices Among Primary Care Providers in Appalachian Pennsylvania

**Published:** 2009-03-15

**Authors:** Eugene J. Lengerich, Nicole L. Huey, Allison D. Clark, ACTION Health Cancer Task Force, Brenda C. Kluhsman

**Affiliations:** Department of Public Health Sciences, The Pennsylvania State University; ACTION Health, Danville, Pennsylvania; ACTION Health, Danville, Pennsylvania; ACTION Health, Danville, Pennsylvania; The Pennsylvania State University, Hershey, Pennsylvania

## Abstract

**Introduction:**

The incidence of cervical cancer in Appalachia exceeds the national rate; rural Appalachian women are at especially high risk. We assessed the attitudes and practices related to human papillomavirus vaccination among providers in primary care practices in a contiguous 5-county area of Appalachian Pennsylvania.

**Methods:**

In December 2006 and May 2007, all family medicine, pediatric, and gynecology practices (n = 65) in the study area were surveyed by 2 faxed survey instruments.

**Results:**

Of the 65 practices, 55 completed the first survey instrument. Of these 55, 44 offered the vaccine to their patients. Forty of the 44 practices offered it to girls and women aged 9 to 26 years, and 11 were willing to accept referrals from other practices for vaccination. The average reported charge for each of the 3 required injections was $150. Of the 55 practices that responded to the first survey instrument, 49 responded to the second survey instrument, 46 of which recommended the vaccine to their patients.

**Conclusion:**

The prevalence of offering the vaccine against human papillomavirus was high in this area of Appalachian Pennsylvania. Future interventions may focus on community education because the vaccine is available from most providers.

## Introduction

Human papillomavirus (HPV), which causes genital warts and cervical cancer, is the most common sexually transmitted infection in the United States; an estimated 6.2 million people are infected every year (1). The prevalence of HPV infection increases with each year of age, from 14 to 24 years, followed by a gradual decline through age 59 (2).

In June 2006, the US Food and Drug Administration licensed HPV quadrivalent vaccine for use in girls and women aged 9 to 26 years as a vaccine against HPV types 6, 11, 16, and 18, which collectively account for 70% of cervical cancers and 90% of genital warts (1). Pediatricians (3) and family physicians (4) reported that they would be more likely to administer an approved HPV vaccine to girls than to boys and to older children than to younger children (5). A systematic, theory-based review of 28 studies conducted before the HPV vaccine was licensed found that parents reacted positively to the possibility of vaccinating their daughters against HPV, especially if they thought infection was likely and the vaccine was effective and recommended by a physician (6).

Cervical cancer incidence and mortality are higher in Appalachia than in the United States as a whole (7,8). ACTION Health is a nonprofit community organization that uses evidence-based initiatives to improve health and eliminate health disparities among residents of a contiguous 5-county (Columbia, Montour, Northumberland, Snyder, and Union) area in Appalachian Pennsylvania (Figure). The 5 counties of ACTION Health are largely rural (52%) and have a median population of 43,387 (range, 17,934-91,654), which is predominantly (96%) white (9). Women and girls aged 15 to 24 make up a median 8.8% (range, 5.3%-12.0%) of the population in these counties, and a median 13.9% (range, 9.6%-14.8%) of the county population is eligible for medical assistance (10).

**Figure.**
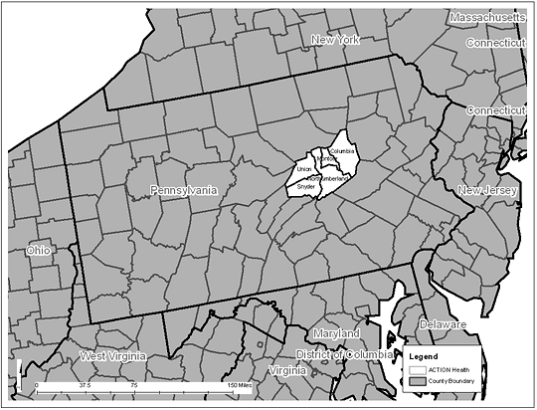
ACTION Health area: Columbia, Montour, Northumberland, Snyder, and Union Counties, Pennsylvania.

From 2003 through 2005, 45 cases of invasive cervical cancer were reported in the 5-county ACTION Health area. According to the Pennsylvania Cancer Registry, 31 cases were expected during this period (11). Also during this period, 12% of women aged 18 to 64 were uninsured and 17% did not have a regular physician (11,12).

Because of the apparent increased risk of cervical cancer in this area, these women may benefit from HPV vaccination. We surveyed primary care practices to determine HPV vaccine–related practices and recommendations in the ACTION Health area. The study was the first of its kind in the ACTION Health service area and the northern Appalachian region overall.

## Methods

In December 2006, ACTION Health conducted an initial survey of all primary care practices (n = 65), defined as family medicine, pediatric, and gynecology practices, including public clinics and university health centers, in the ACTION Health area. We identified practices by reviewing telephone listings and hospital Web sites and by querying coalition members. We initially contacted practices by telephone, and then we faxed them a 1-page survey (Appendix) that took less than 5 minutes to complete. We asked the person in the practice who was most knowledgeable about the HPV vaccine to complete and return the instrument.

In May 2007, a second survey was faxed to the people who returned the initial instrument (n = 55) because we wanted to develop a strategy for a future intervention (Appendix). The second instrument took approximately 5 minutes to complete. We did not collect data on the training or position of the person who completed either instrument. We calculated prevalence estimates and 95% confidence intervals (CIs) for all results. The institutional review board of The Pennsylvania State University determined this study to be exempt from review.

## Results

Of the 65 primary care practices, 55 returned a completed initial instrument (response rate, 85%). Of these, 44 (80%; 95% CI, 69%-91%) offered the vaccine to their patients. Fifteen (27%; 95% CI, 16%-39%) practices that offered the vaccine reported at least 1 concern about the vaccine; concerns included cost and insurance coverage (n = 9), newness of the vaccine (n = 7), that the recommended age was too young (n = 4), and not knowing what type of physician should administer the vaccine (n = 1). Of the 44 practices that offered the vaccine, 40 (91%) offered it to all girls and women aged 9 to 26 years; 11 (25%) reported that they were willing to take referrals of other practices' patients for vaccination, although most reported that a new patient must first receive a comprehensive exam and related care (for example, Papanicolaou test). The average reported charge for each of the 3 required injections was $150, which included the cost of the office visit. Thirty (68%) respondents reported that they were willing to post flyers on HPV educational programs. Of the 11 practices that did not offer the vaccine, 9 (82%) expressed interest in offering it in the near future, and 2 (18%) requested additional information. Reasons that the 11 practices gave for not currently offering the vaccine included planning to offer the vaccine in the near future (n = 3), more research and information needed (n = 2), vaccine too new (n = 2), not enough patient interest (n = 1), physician at the practice had been ill and had no information (n = 1), vaccine should be given by the patient's gynecologist (n = 1), and needed corporate approval to offer the vaccine (n = 1).

Of the 55 practices that responded to the initial instrument, 49 (89%) completed the second instrument. Of these, 46 (94%; 95% CI, 87%-100%) reported that they recommended the vaccine to their patients, and 44 (90%; 95% CI, 81%-98%) reported that patients were requesting the vaccine for themselves or their daughters. Eighteen (39%) practices reported mostly vaccinating only those younger than 18 years, 10 (22%) reported mostly vaccinating only those aged 18 to 26, 11 (24%) reported vaccinating both age groups, and 7 (15%) did not report a specific age group. Several practices reported that they did not vaccinate girls in certain age groups: rarely under 11 (n = 1), not under 12 (n = 1), not under 13 (n = 1), not under 15 (n = 1), and not under 16 (n = 2). In some cases, practices indicated that the approved age was too young, and in other cases they indicated they had no requests for the vaccine in those age groups. The 46 practices that recommended the vaccine gave an average of 39 (range, 33-45) first doses each week; 41 practices (89%) reported that patients received the second and third doses. Six (13%) providers who recommended the vaccine were willing to speak at a future educational program. Practices that recommended the vaccine reported concerns, including cost (n = 2; 4%) and limited patient interest because the vaccine was relatively new (n = 1; 2%). Of the 3 practices that did not recommend the vaccine, 2 were concerned that the recommended age was too young, and 1 reported a concern about the vaccine's safety and effectiveness.

## Discussion

We found that the HPV vaccine is being offered to patients in the approved age range, 9 to 26 years, in a 5-county region of Appalachian Pennsylvania. Most practices that did not currently offer the vaccine intended to offer it in the future. In addition, most practices were willing to provide the vaccine to patients of other practices and participate in educational programs. Barriers to vaccination included the cost of the vaccine, especially for women aged 18 to 26 years, an age group that is not covered by the Vaccines for Children program. The belief among some providers that the recommended age is too low and that the vaccine promotes sexual activity among young women may also restrict access to the vaccine. One family health practice that did not offer the vaccine at the time of the initial survey ("We are doing our own research and looking to order vaccine soon.") reported on second survey that they were offering the vaccine but not to young girls ("[The vaccine] is not given to the extremely young. We are waiting for the vaccine to prove effective and safe. I don't want patients to feel falsely safe and free to sleep around.").

These findings can guide future community-based initiatives to increase vaccination. Barriers to vaccination, including cost and health insurance coverage, should be addressed in future initiatives. Other barriers have previously been reported, including lack of knowledge about HPV transmission and associated risks for cervical cancer and other genital diseases, parental concerns about the vaccine and vaccinating minors against sexually transmitted infections, lack of knowledge about financial assistance programs, challenges related to health care–seeking behavior of young women, and barriers specific to HPV infection (eg, protection against only certain types of HPV) (13).

Few evidence-based interventions to increase vaccination have been reported. A randomized study conducted before the HPV vaccine was licensed found that parents who received an HPV information sheet had higher mean scores on HPV knowledge than did parents who did not receive the information (13). However, the groups did not differ in terms of vaccine acceptability (13). Among women aged 18 to 30, knowledge, personal beliefs about vaccination, belief that others would approve of vaccination, and a higher number of sexual partners were significantly associated with intention to vaccinate (3). A study of women aged 18 to 25 found that those who had had vaginal sex in the past 12 months were 4 times as likely to report acceptance of vaccination (14). In addition, those who had ever had a sexually transmitted infection or an abnormal Papanicolaou test were also more likely to indicate acceptance.

Limitations to this study must be acknowledged. The sample was drawn from a specific geographic area, thus limiting the generalizabilty of the results. Several factors may have limited the validity of the results. First, the study collected data that were self-reported and not verified by direct observation or medical record review. The study also used 2 instruments that had not been validated previously. Instruments that had been validated in previous studies would have been preferable, but none was known to the investigators as the study was being planned. Two surveys of health care providers have since been reported (15,16).

The study also has numerous strengths. First, the response rate was high, which may be partially because the survey was brief, was conducted by a local organization, and focused on a timely topic. Second, the survey was conducted in Appalachia, a region that has cervical cancer health disparities. Third, the study was participatory and initiated communication between ACTION Health and local primary care practices. Future partnerships may develop from this initial communication, which could expand ACTION Health's network of health care affiliates for cancer prevention and control. Finally, the study design was community-based, and results can be used to facilitate future interventions in the specific area of ACTION Health.

We conclude that most practices in this area of Appalachian Pennsylvania recommend the HPV vaccine or provide it if patients request it. Future community education interventions should target women and girls in the appropriate age range to increase their knowledge of cervical cancer and the risks and benefits of HPV vaccination.

## Appendix

### Initial 1-page survey on human papillomavirus vaccine faxed to all primary care practices in the ACTION Health area (5 rural counties in Appalachian Pennsylvania)

Recently, there has been growing interest in the human papillomavirus (HPV) and its relation to cervical cancer. In 2006, the US Food and Drug Administration approved a vaccine to prevent cervical cancer caused by HPV. Gardasil, developed and produced by Merck Pharmaceuticals, protects against the 4 main types of HPV. Family planning centers and the Pennsylvania Department of Health have had numerous requests for the vaccine and information about the vaccine. Currently, the Pennsylvania Department of Health does not offer this vaccine. The ACTION Health Cancer Task Force is conducting a survey of all family practice centers, obstetricians/gynecologists, and pediatricians to determine the availability and present use of this vaccine. By conducting this survey, the Cancer Task Force hopes to offer educational programs to help better educate the public about HPV, cancer, and the vaccine. Thank you for completing this short survey.

**Table:**

1.	Do you currently offer the HPV vaccine? (yes/no)
	**If yes:**
	Do you offer the vaccine to all females ages 9 to 26? (yes/no)
	If no, please explain.
	Do you accept referrals for non-patients interested in receiving the vaccines? (yes/no)
	How much do you charge for the vaccine for the uninsured or underinsured?
	**If no:**
	Are you interested in offering this vaccine? (yes/no)
	If no, please explain.
	Would you like more information about this vaccine? (yes/no)
2.	Would you be willing to post flyers in your medical practice about HPV educational programs being offered by the ACTION Health Cancer Task Force? (yes/no)
3.	Please list any concerns, barriers, or comments you have about the HPV vaccine.

#### Follow-up survey to primary care practices that responded to the initial survey

Recently, there has been growing interest in the human papillomavirus (HPV) and its relation to cervical cancer. In December 2006, ACTION Health Cancer Task Force surveyed local family practice centers, obstetricians/gynecologists, and pediatricians to see if the HPV vaccine was available, any insurance concerns, and any general concerns about this new vaccine. The response to the survey was wonderful; 85% of those surveyed responded. Because of the responses from the surveys we have been able to offer several education programs about HPV and cervical cancer. We are now asking you to please complete our follow-up survey. This short follow-up will help us learn more about our counties' response to the HPV vaccine. Thank you for completing this short survey.

**Table:**

1.	Are patients requesting the vaccine for themselves or for their children?
2.	Are doctors and medical staff recommending the HPV vaccine to their patients? (yes/no)
	**If yes:**
	Do they recommend it for all females ages 9 to 26? (yes/no)
	If no, please explain.
3.	On average how many initial (first dose) HPV vaccines does your practice administer in a week?
4.	Are patients keeping their follow up appointments for the second and third dose? (yes/no)
5.	Do you administer mostly to girls ages 9 to 17 or women ages 18 to 26?
6.	Would you like more information about this vaccine? (yes/no)
7.	Would any of the doctors or medical staff be interested in being a guest speaker for an educational program about HPV and cervical cancer? (yes/no)
8.	Please list any concerns, barriers, or comments you have about the HPV vaccine.
